# Sensory-specific impairment among older people. An investigation using both sensory thresholds and subjective measures across the five senses

**DOI:** 10.1371/journal.pone.0202969

**Published:** 2018-08-27

**Authors:** Annachiara Cavazzana, Anja Röhrborn, Susan Garthus-Niegel, Maria Larsson, Thomas Hummel, Ilona Croy

**Affiliations:** 1 Smell & Taste Clinic, Department of Otorhinolaryngology, TU Dresden, Dresden, Germany; 2 Gösta Ekman Laboratory, Department of Psychology, Stockholm University, Stockholm, Sweden; 3 Department of Psychotherapy and Psychosomatic Medicine, Faculty of Medicine of the Technische Universität Dresden, Dresden, Germany; 4 Department of Child Health, Norwegian Institute of Public Health, Oslo, Norway; University of Ottawa, CANADA

## Abstract

Age-related sensory impairment is a slow and gradual progress, which affects multiple modalities. Two contradictory hypotheses exist about the age-related decline of sensory thresholds. The **common factor theory** assumes one underlying factor—which accounts for the loss of several sensory modalities simultaneously—and the **specific factor theory** predicts that the sensory decline is uncorrelated between different modalities. In this study, we aimed to explore whether (i) there is a common factor of sensory thresholds in older people, (ii) older people assume that sensory decline in one modality also affects other modalities, (iii) there is a relation between sensory threshold and the subjective assessment of sensory function. This was accomplished by collecting both threshold measures and self-reported ratings for smell, hearing, taste, vision, and touch function in a group of 104 older people (mean age: 67.2 years; SD: 9.85; range: 50–100 years). Results indicated that there was no common factor of sensory thresholds, hence an impairment in one modality did not necessarily imply a shortfall in other modalities. In contrast, our results suggested one or two common factor(s) for the participants’ ratings. Participants who reported a diminished function in one sense tended to generalize this rating to the other senses as well. The correspondence between subjective ratings and sensory thresholds was relatively good for vision and audition, although no correlations were observed for the other domains. These findings have implications for clinicians, suggesting that subjective measures should be combined with sensory threshold measurements when evaluating sensory dysfunction. Also, these data convey a positive message for older people and their physicians by showing that loss in one sensory modality does not necessarily generalize to losses across all sensory modalities.

## Introduction

As we age, our sensory functions decline contributing to an increased isolation from the outside world, forcing us to adopt perceptual aids (e.g., glasses, hearing aids). This has been demonstrated especially for hearing and vision (for a review see: [1]). Older people usually experience a decline in visual acuity because of changes in lens elasticity which consequently lead to a decrease in abilities to focus on near objects (i.e., presbyopia) and to adapt to light [2]. Also hearing is well known to decline with age [3] and is usually characterized by a decreased hearing sensitivity, capability to understand speech in a noisy environment, slowed central processing of acoustic stimuli, and impaired sound localization. It has been estimated that about 30% of men and 20% of women in Europe have a hearing loss of 30dB or more at the age of 70 years old [4]. Similarly, deficits in smell and taste are highly prevalent in older people from approximately 60 years of age [5–7]. Reports for the somatosensory systems are less consistent than for hearing, vision, taste and smell [8], but researchers have nevertheless reported a significant age-related decline in vibrotactile sensitivity as shown by increase in threshold for perception of vibration [9–11].

Impaired sensory functioning impacts the quality of life of older people [12] by influencing the way they experience the environment and react to stimuli and limiting social activities—facts which may lead to isolation and depression [13,14]. Sensory deficiency has also negative consequences for somatic health. For example, vision loss increases the risk of falls and fractures [12], whereas olfactory loss complicates the detection of dangers in the environment (e.g., smoke, gas, spoiled food [15]) and may lead to changes in food choice. Although age-related decline in each of these sensory systems has been well established in the literature, little is known about whether these systems tend to be impaired concomitantly, as researchers have usually focused their attention at one sensory modality at a time or only used self-reports [16–18]. Furthermore, different quantitative methods have been adopted when investigating multiple sensory impairments, ranging from sensory threshold to supra-threshold measures [12,19–21]. Out of those, threshold testing seems to be the best method to explore age-related sensory loss as they reflect mainly peripheral processes and are less related to higher order cognitive processing (e.g., memory functions). This is advantageous for older people, where cognitive impairments may become an issue.

In younger individuals, no general factor of sensitivity is observed. Hence, there is no correlation between olfactory, trigeminal pain and gustatory thresholds [22]. In older individuals, Stevens et al. [23] were–to the best of our knowledge—the first to compare more than two modalities directly using threshold methods. They measured sensitivity thresholds for taste, smell, thermal sensitivity (cooling), vibrotaction (both low- and high-frequency), and hearing (both low- and high-frequency) in 15 young and 22 older participants and observed an age-related reduction in sensitivity and acuity. The authors found significant positive correlations between all the modalities and age accounted for most of the variance challenging the “common factor theory” (or global sensory impairment) which instead postulates that common mechanisms underlie the decline of the sensory systems, supporting the concept of a unified underlying process of sensory aging [20,24]. Such a framework is conceptually similar to the concept of the “common cause hypothesis”, which states that a common underlying factor drives age-related deterioration in cognitive and sensory processes [23,25,26]. In literature, support for this hypothesis is mixed and it is usually due to an artifact of the research design, i.e., to the pooling of data from measures across extremes of the adult age continuum, which can inflate the correlations [27,28]. Indeed, this somewhat pessimistic prediction for older people was also not supported by later studies which tried instead to reduce such methodological issue. For example, Humes et al. [27] examined differences between 42 young and 137 older people for auditory, visual, and tactile measures of threshold sensitivity and temporal acuity (gap-detection threshold) and found, again, an effect of aging on these measures. They observed only very few cross-modality sensory associations in older adults, supporting the “specific factor theory”, which states that each sensory system undergoes a distinct age-related degeneration [26,27,29–32]. In line with this, Gadkaare et al. [33], testing four sensory systems in 276 people, also demonstrated that aging caused a shortfall of several sensory functions (vision, touch, audition, and vestibular function), but through mechanisms which seem unique to each sensory system. Taken together, the common factor hypothesis seems not to be supported by these abovementioned studies [23,27,33] and was replaced by the hypothesis of sensory specific factors.

All three studies neglected the self-report dimension. Hence, it is unclear whether older people assume that sensory decline in one modality also affects other modalities, and whether there is a relation between sensory threshold and the subjective assessment of sensory function. The self-report dimension is however important in order to understand how older people perceive their own sensory abilities. Age-related sensory loss in healthy people is usually a slow and gradual process which leads to a reduced awareness of sensory deficits by older people [34–36] and therefore, self-report and threshold measurement may not align well. To the best of our knowledge, there are no studies that have explored whether there is a subjective global sensory impairment considering all the five senses together. The ones that have explored the association between the self-reported sensory impairment and the subjective well-being usually considered only vision and hearing and found an association between the two measures [37–39].

Understanding whether the sensory systems tend to lose functioning simultaneously (or follow an independent route of decline) and whether people are aware of this decline will help clinicians and caregivers to achieve a better comprehension of physical and psychosocial challenges experienced by those people suffering from this loss. Addressing the sensory impairment will ensure adequate quality of care, limiting the impact on the daily life functioning. This will also be of growing importance for rehabilitation services and others who work in the fields of sensory loss and aging, as well as for those affected by this condition in order to maintain the functional independence of older people and to develop rehabilitation models that address multiple impairments. In addition, knowing the awareness of these changes will determine whether or not older people can make adaptive changes as a consequence of such loss.

The main purpose of the present study was to test directly sensory thresholds and self-assessment of several modalities (i.e., smell, hearing, taste, vision, touch) in the same group of older people. We hypothesized, in line with the specific factor theory, that no common factor of sensory functioning can be found across threshold measurements of different senses. For the subjective perception, we aimed to explore whether such a common factor exists. Furthermore, we expected to replicate the negative correlation between age and sensory function and we assumed a lack of significant correlations between sensory threshold measures and subjective ratings, reflecting the limited awareness of sensory loss.

## Methods

### Study design and participants

This cross-sectional descriptive study included a total of 104 participants (69 females; mean age: 67.2 years; SD: 9.85; range: 50–100 years) took part in the study. Subjects were recruited via public advertisement postings from the Smell and Taste Clinic which were placed in various locations on the Medical Campus. The selection criteria were based on age (≥ 50 years old) and—in order to rule out a potential cognitive impairment—a score ≥ 27 on the Mini Mental State Examination was set [40]. In addition, patients with chronic disease (e.g., Alzheimer's disease, Parkinson's disease, diabetes) were excluded from this study. The vast majority of the participants self-reported to be in good general physical and mental health (Table 1). None of the participants reported to be severely depressed. All aspects of the study were compliant with the Declaration of Helsinki. The Ethics Committee of the Medical Faculty at the TU Dresden approved the study (application number: EK116042013). All the participants were informed about the general procedure of the experiment and signed a written consent form. Each subject underwent sensory evaluation as detailed below.

**Table 1:** Demographic and health characteristics of the sample.

Age (mean; SD)	67.2; 9.85
MMSE (mean; SD)	29.5; 0.7
Female %	69
Head injuries %	11.5
Frequent nasal sinuses %	6.7
Hay fever %	10.6
Headache %	17.3
Hyperthyroidism %	3.8
Hypothyroidism %	7.7
Frequent colds %	4.8
Nasal polyps %	3.8
Nasal breathing problems %	9.6
Rhinorrhea %	21.2
Snoring problems %	41.3
Liver inflammation %	8.7
Renal diseases %	6.7
Nasal cave surgery* %	3.8
Nasal septum surgery* %	8.7
Nasal polyps* %	1.9
Palatine tonsil surgery* %	15.4
Pharyngeal tonsil surgery* %	22.1
Middle ear surgery* %	4.8
Oral surgery* %	28.8

*None of the surgeries happened less than 6 months prior testing

### General procedure

Demographic variables, questions about their self-perceived sensory functions (see the paragraph named “Questionnaires”), medical history and the cognitive status were investigated. Subsequently, participants underwent a comprehensive set of threshold tests in the following fixed order: smell, hearing, taste, vision, touch (see below for details). Most of the participants (66.1%) were tested in the laboratory of the Smell and Taste clinic, the other 33.9% at their respective home. Trained interviewers administered detailed questionnaires on demographic and health-related data and the sensory tests. Regarding the sensory threshold and the subjective ratings, data were collected for all the participants (N = 104). One missing answer was reported by one of the participants regarding the taste self-ratings (N = 103).

#### Threshold measurements

The choice of tests was guided by a simple and transportable assessment (as some of the measurements took place at the participant’s home). Wherever possible, we used a multiple forced-choice staircase procedure for sensory testing, which provides reliable results [41]. This was the case for the thresholds of hearing and smell. However, in order to prevent fatigue effects, which are common in older people [42], consideration was taken to keep the overall test time below 90 minutes. We therefore compromised and used a multiple forced-choice ascending threshold procedure for the sense of taste and a (no forced choice) ascending threshold procedure for vision and touch. For the measurements of touch, taste, and smell, a visual shield was used to prevent visual distraction.

**Olfaction.** Olfactory thresholds were assessed birhinally with the “Sniffin' Sticks” test [43]. Odors were presented in felt-tip pens. The olfactory detection threshold task consists of 16 dilutions starting from 4% phenyl ethyl alcohol solution (PEA, rose-like odor; ratio 1:2 in deionized water). During the test, one pen at a time was presented in front of the nostrils at a distance of approximately 1 to 2 cm: two pen sticks with a solvent and one pen stick with the PEA. The experimenter asked the participants to detect the pen stick containing the PEA in each trial. The pen sticks were presented for 5 s, and the inter-stimulus interval was 10 s. After correct identification of two successive trials, the examiner presented a new trial with descending concentration of the odor. When the participant misidentified the odor, the examiner presented a sequence of odor with ascending concentration. The test ended as soon as a series of seven descending and ascending trials were completed. We computed the olfactory threshold as the mean of the last four out of seven staircase reversals. The scores ranged from 16 (participants being able to detect the lowest concentration) to 1 (participants being unable to detect even the highest concentration).

**Audition.** Auditory thresholds were measured for pure-tone frequency (i.e., 4000 Hz), which is in the domain of speech and therefore ecologically relevant. The tones were generated by an audiometer (MA-11_Screening-Audiometer–Präcitronic, Dresden, Germany) including headphones. Each ear was tested first individually, and then together; it always started with the presentation of the tone at 0dB to the right ear. Then the volume was regulated up to a starting position clearly perceivable by the participant. From there, the test started. The experimenter indicated the numbers “one”, “two”, and “three” with his/her fingers and presented the tone at only one of the occasions. The participant reported the number at which he/she perceived the tone. If the participant was able to correctly perceive the sound twice in sequence, the sound level was lowered by 5 dB. If the participant made a mistake, the sound level was increased by 5dB. For each auditory test, five turning points were performed and the threshold was defined as the mean of the last four. The raw scores ranged from -3 (best performance) to 100 (worst performance).

**Gustation.** Gustatory thresholds were assessed for sweet (sucrose) stimuli. Administration of the taste stimuli was based on the principles used with the “Taste strips” [44] with 1 cm^2^ of filter paper being impregnated with a tastant. The dried filter papers were then applied to the tongue. The strips were impregnated with a maximum of 10 dilutions starting from concentrations of 80 g sucrose in 120 ml water. Dilutions were made in geometric series of 1:2. The participant received three strips, one after each other on the middle of the tongue, with only one containing the tastant. The participants’ task was to determine the strip containing the taste. Then, after each triplet presentation, subjects rinsed their mouth with fresh tap water. Triplet presentation started with the lowest concentration and stopped when the participant correctly identified both triplets of the same concentration. We computed the gustatory threshold as the mean of the last two out of ten staircase reversals Scores ranged from 1 (participants being unable to detect even the highest concentration) to 10 (participants being able to detect the lowest concentration).

**Vision.** Near vision was measured using a standard reading table presented at reading distance (30 cm). Participants had to read 10 simple sentences, which varied in content and font size (font: Times New Roman; size: 18,14,12,11,10,9,8,7,6,5). They started to read the first sentence using the largest font size (i.e., 18) without visual aids. The last step/font size, which could be read without errors, corresponded to the threshold for close-up viewing. Afterwards, participants who usually wore glasses in their daily lives repeated the whole test (i.e., a parallel version with different sentences) with their visual aids. For statistical analysis, we focussed on visual perception without compensation (i.e., without glasses), as the other senses were as well tested without any technical augmentation. The score ranged from 0 to 10, hence a participant who was able to read the front size 14—but not 12—had a score of 2; a participant who was able to read even font size 5 had a score of 10.

**Touch.** Vibration threshold was determined with a 64Hz tuning fork, designed for vibration assessment [45]. For this purpose, the fork was placed on a bone point on the distal forearm, i.e., the processus styloideus ulnae. Participants were instructed to concentrate on the vibration and to communicate to the examiner when he/she could no longer perceive it. A scale on the tuning fork showed a value between 0.5 (only large amplitudes are perceived) and 8 (perceptions of small amplitudes) with markings every 1/8. The normative value for healthy individuals is reported at 8/8. The value diminished with subsiding vibration and hence a high value corresponded to a sensitive perception of vibration stimulations. Importantly, the assessment does not produce audible tones, but vibration only. The test was repeated three times and the arithmetic mean of all three values resulted in the vibration threshold.

#### Questionnaires

The paper-and-pencil questionnaires were directly completed by the participants, who were asked to self-report their sensory function compared to peers of the same age and compared to past ability (i.e., when they were younger). For example, self-reported hearing was evaluated with the following question: “How is your audition compared to other people of the same age?”; (ii) “How is your audition compared to when you were young?” with response categories of 1 “very good”, 2 “clearly better”, 3 “slightly better”, 4 “normal”, 5 “slightly decreased”, 6 “clearly decreased”, 7 “very bad”. Self-reported vision, smell, taste and touch were evaluated using the same questions and scales.

### Statistical analyses

Analyses were conducted in the framework of structural equation modeling [46] and performed using the statistical program Mplus 8 [47] as well as SPSS software version 21.0 (SPSS Inc., Chicago, IL, USA). All analyses were tested at a significance level of 0.05. First, the sensory threshold measurements of the sensory functions were examined. In order to directly compare the raw threshold measures obtained across modalities, the raw score for each measure was transformed into z-scores.

In order to investigate whether the sensory threshold impairment was sensory-specific, we conducted confirmatory factor analyses of the threshold measurements of the five sensory functions, trying to construct a latent factor for global sensory impairment. For the subjectively rated sensory functions, we first described the percentage of participants rating their respective sensory functions as above or below normal functioning. Similar to the threshold measurement, we conducted confirmatory factor analyses of the subjective ratings of the five sensory functions, trying to construct a latent factor for subjective global sensory impairment. We focussed on ratings “in relation to peers” as this rating is less biased by a memory effect. Then, the relationships between age and threshold measurement were assessed. Bonferroni corrections for multiple comparisons adjusted for dependent measurements have been performed for all correlations.

## Results

Demographic and health characteristics of participants are presented in Table 1. Raw and z-score data for each sensory modality were reported in Table 2.

**Table 2:** Means (SD) for raw and z-score data.

	Mean—raw score	Mean—z-score
Smell	7.69 (3.64)	0.014 (1.05)
Audition (both ears)	64.53 (24.02)*	0.066 (1.03)*
Taste	8.52 (1.67)	0.0094 (1.04)
Vision	5.36 (3.54)	0.028 (0.98)
Touch	7.63 (0.53)	0.11 (0.87)

*Scores regarding audition were reversed to make it congruent with the other tests. In this way, threshold values were all expressed in the same direction and higher means indicated a better performance.

**Fig 1:**
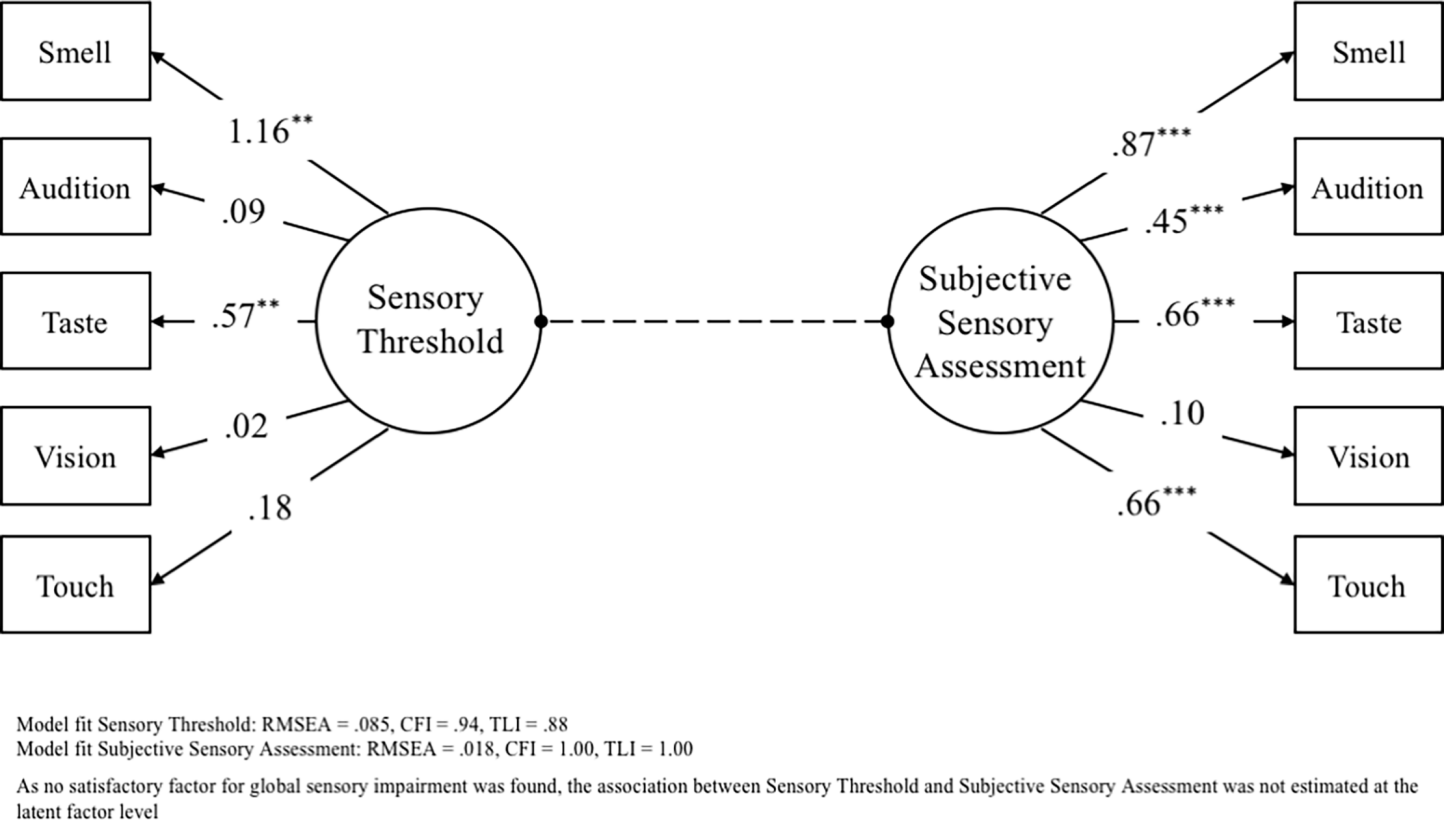
Visual representation of the model and factor loadings for the one-factor solutions.

First, we tried to conduct a confirmatory factor analysis of the **threshold measurements** of the five sensory functions with a one-factor solution. The results showed a suboptimal fit [root mean square error of approximation (RMSEA) = 0.085, comparative fit index (CFI) = 0.94, Tucker–Lewis index (TLI) = 0.88], with fit indices outside the recommended range (48). Most factor loadings were rather small and three out of five (audition, vision, and touch) not even statistically significant (see Fig 1). Based on both the factor loadings and the thresholds’ intercorrelations we then tested a two-factor solution, similarly yielding a suboptimal fit (RMSEA = 0.088, CFI = 0.95, TLI = 0.88) Further, in order to compare both models we conducted a Chi-Square Difference Test, yielding a non-significant χ2 of 1.577 with 1 degree of freedom (df), i.e., a two-factor solution did not fit the data significantly better. Therefore, we concluded that, in line with our hypotheses, sensory threshold impairment among the older people in our sample was sensory-specific and not due to global sensory impairment.

**Fig 2:**
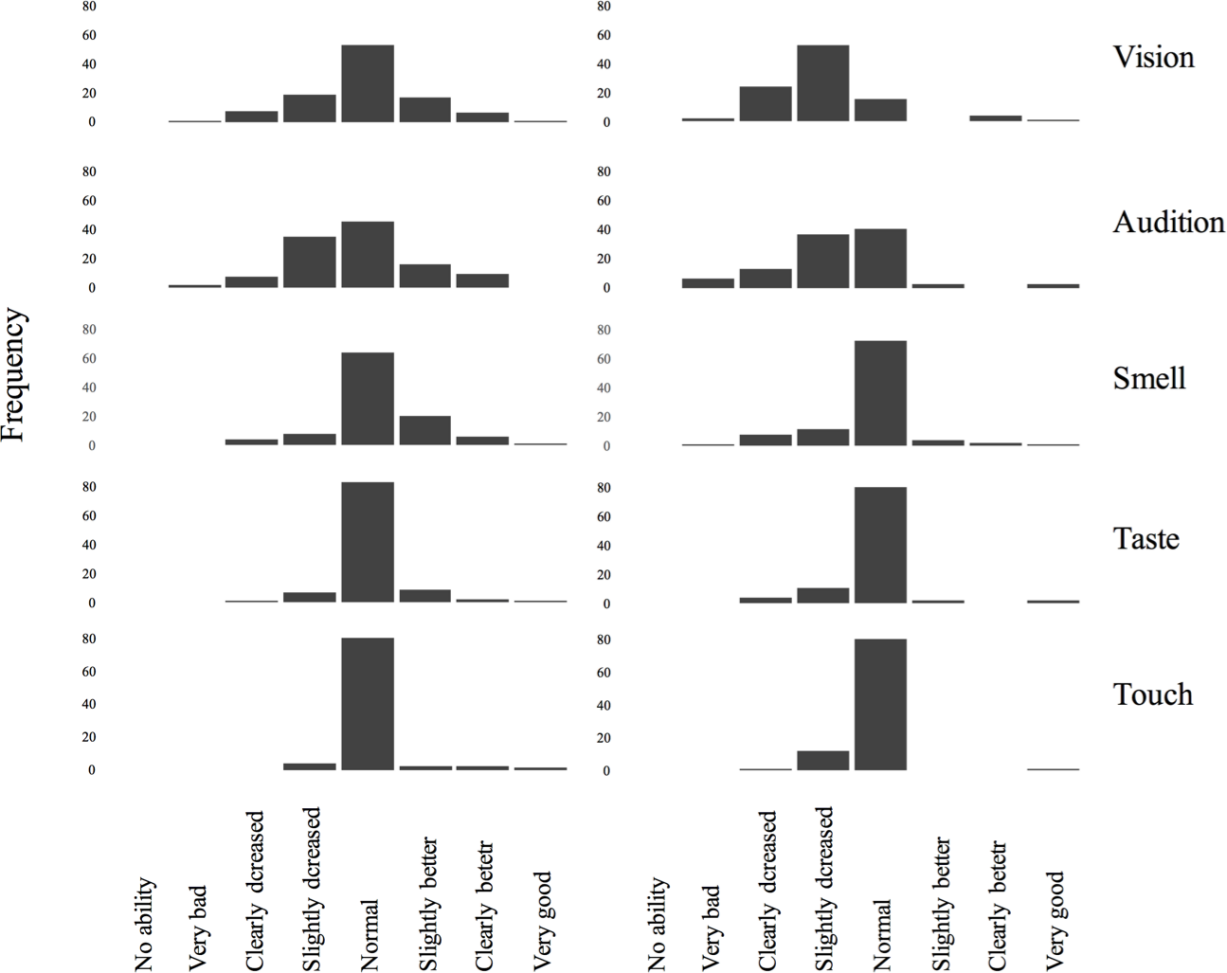
Ratings of sensory functions in relation to age peers (left) and in relation to young age (right).

Regarding the **subjective ratings** of the five sensory functions, the majority of participants reported “normal” sensory functions in *relation to peers* (Fig 2, left): 32.6% reported subjective impairment for audition, 26% for vision, 11.5% for smell, 7.8% for taste, and 3.8% for touch. The confirmatory factor analysis showed a very good fit for a one factor solution, when allowing a correlation between the residuals of smell and touch (RMSEA = 0.018, CFI = 1.00, TLI = 1.00). Unlike the threshold measurements, most factor loadings were satisfactory and only vision was not statistically significant. Despite the very good model fit, we also tested a two-factor solution, yielding a slightly worse but still good fit with fit indices within the recommended range [48] (RMSEA = 0.043, CFI = 1.00, TLI = 0.98). Next, we conducted a Chi-Square Difference Test, yielding a non-significant χ2 of 0.544 with 1 df, i.e., statistically the model fit of the two-factor solution was not significantly different.

In relation to the *past* (Fig 2, right), participants surprisingly often still perceived their sensory functions as normal, except for vision and audition, where the peak was shifted to “slightly decreased”. Here, the percentage of impairment was higher as compared to ratings in relation to peers: 79% reported an impaired sense of vision, 56% of audition, but only 21% reported an impaired sense of smell, 15.1% of taste, and 13% of touch.

**Fig 3:**
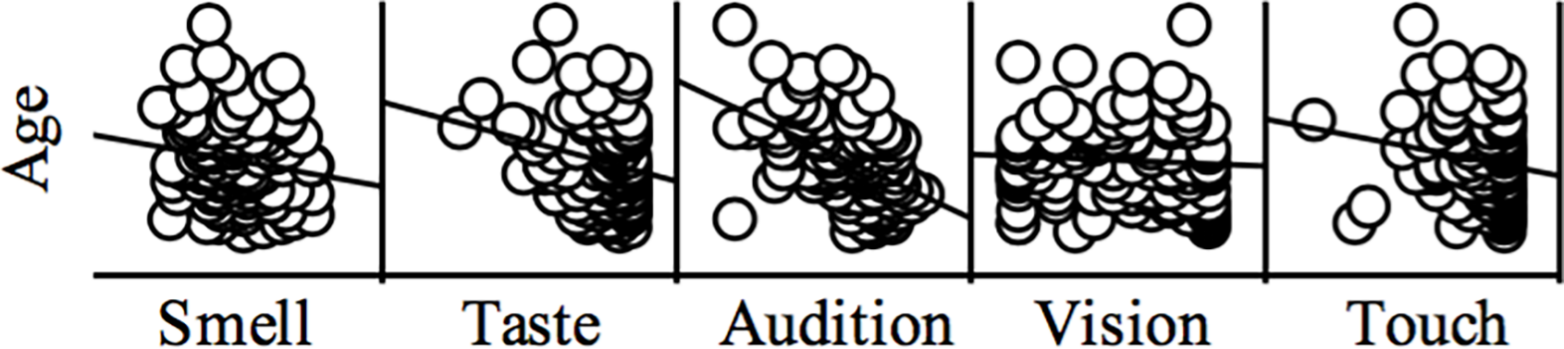
Scatterplots depicting the sensory thresholds (z-score) in relation to age. Scores regarding audition were reversed to make it congruent with the other tests. In this way, threshold values were all expressed in the same direction and higher means indicated a better performance.

As no satisfactory factor for global sensory impairment was found, **the relationship between age and sensory impairment** was estimated separately for each sensory function. A significant correlation between participants’ age and auditory threshold (*r* = 0.49, *p*_*corrected*_ < 0.001) was observed. Furthermore, a negative relationship was observed also between age and taste (*r* = -0.24, *p*_*uncorrected*_ = 0.016), but this correlation did not hold for corrections for multiple measurements. Chronological age was unrelated to the measure of non-compensated vision (*r* = -0.06, *p* = 0.51), touch (*r* = -0.17, *p* = 0.084), and olfactory threshold (*r* = -0.16, *p* = 0.11). For an overview, see Fig 3.

As our results indicated no global sensory impairment, we estimated the **relationship between sensory thresholds and subjective sensory functions** using Spearman’s correlation coefficients. As shown in Table 3, the threshold functions and the subjective sensory assessment correlated moderately well for vision and audition, but not for the other senses.

**Table 3:** Correlations between sensory threshold and self-report ratings of sensory functions.

Sensory function	subjective ratings in relation to peers				
	vision	audition	smell	taste	touch
Vision	-0.27**	-0.16	-0.09	-0.11	-0.07
Audition	0.11	-0.46**	0.19	-0.05	-0.04
Smell	-0.02	0.03	-0.01	-0.13	-0.17
Taste	0.1	0.02	-0.14	-0.03	0.09
Touch	-0.11	-0.26**	-0.18	-0.03	-0.12

** *p*_*corrected*_ < 0.001

## Discussion

In this study we explored whether there is a common factor of sensory threshold or subjective assessment in older participants. We furthermore explored the relationship between sensory thresholds and subjective assessment in order to examine participants’ awareness of their sensory functions.

Results from the confirmatory factor analysis of the threshold measurements of the five sensory functions indicated that the older people in our sample were not affected by global sensory impairment. Therefore, the results obtained in the present study do not support the common factor hypothesis [20,24–26] which proposes that multiple sensory modalities appear to decline concurrently, reflecting a general neurophysiological decline. In contrast, our results favour the specific factor theory [23,26,29,30,32,49,50], which assumes distinct causes for sensory decline in each domain. The reasons for sensory decline–as outlaid in this theory—are for instance antioxidant levels and vitamin deficiencies which may disproportionately contribute to vision loss, while noise exposure and ototoxic medications may uniquely affect hearing [33].

At odds with a bulk of evidence that report a lower sensitivity with increasing age (e.g., smell: [5,6,51], vision [27,52], taste: [53,54], touch: [8], audition: [55]), no significant correlations were found between age and objective measures of smell, vision, taste, and touch. Only auditory thresholds significantly changed with age in our sample. Age-related changes of sensory sensitivity are grounded on multiple causes: changes on the receptor level, reduced regeneration of peripheral function [56], cortical changes especially in associative areas [57] or a simple lack of practice which may moderate the aforementioned effects. The heterogeneity of a sample of older participants, however, leads to a considerable variation of health and experience. For instance, older people, who train their sense of smell, have a considerable better olfactory threshold [58]; similarly, older people who train three-ball cascade juggling have a cortical thickening in the visual cortex [59]. We tried to reduce this source of noise partly by including only participants with good subjective health. However, this caused a specific sample selection, which was potentially skewed to particularly fit individuals. Another reason for a lack of age-related decline in some of the sensory tests may by the selection of sensory test. For the assessment of touch and taste, ceiling effects were observed which reduced the variability of the dependent variables. This may have prevented the occurrence of a significant relationship with age. For touch, intrinsic structural changes of the skin take place during aging leading to physiological changes that affect the skin's ability to function as an interface between the internal and external environments [60,61]. In particular, the decreased density and function of Pacinian corpuscles is thought to form the basis for age-related declines in vibrotactile sensitivity [62,63]. However, age-related declines of touch perception are not consistent and rather small [8]. Furthermore, this perception varies according to the type of measures used (threshold *vs*. supra-threshold), the modality of the utilized stimuli (warm *vs*. cold), the stimulation site (i.e., distal *vs* proximal), and skin morphology on the stimulation site (i.e., collagen elastin, glabrous vs non-glabrous, subcutaneous fat which influence the resilience of the skin in response to the tactile stimulus) [61,64,65]. For taste, a small relationship to age was found which did not hold for multiple comparisons. The effects of aging on the taste system are quality-specific and sucrose perception is less influenced by age than the perception of bitter for instance [66,67]. Another source of noise in our data might be caused by medication as the extent of taste loss is greater for individuals who consume prescribed drugs [66]. We did however not ask our participants to list all the medications taken. For the odor threshold, scores observed in the present sample (mean: 7.69) were slightly higher than normative data obtained in the same age group (i.e., >55 years old; mean: 7.3) [5], however the sensitivity was lower than the normative olfactory threshold of younger people (i.e., between 36 and 55 years old; mean: 8.75). Finally, regarding vision it might be that the distance we adopted between the participants and the sentences to read was too close (30 cm); therefore the task might have been too easy for them.

For subjective ratings, participants often perceived their sensory functions in relation to the past as normal, except for vision and audition, where the peak was shifted to “slightly decreased”. This was confirmed by ratings in relation to peers, where we found a higher percentage of people reporting an impairment regarding audition and vision. Smaller percentages were observed for taste, smell, and touch. Interestingly, indicated by a very good model fit, the confirmatory factor analysis suggested global sensory impairment when people are asked to self-evaluate their sensory performance in relation to peers. This was different from the threshold measures where only very few associations were found between the various sensory systems. However, also a two-factor solution fitted the data well. Still, in contrast to the threshold measures, the results of the confirmatory factor analyses of the subjective ratings did not suggest entirely sensory-specific impairment. This result might be related to the intrinsic nature of self-report measures: when participants are explicitly asked to think about their sensory functioning, a halo-effect may superimpose their ratings, hence the assessment of one sense is transferred to the other senses.

When correlating the sensory thresholds and the subjective ratings, the subjective assessment of vision and audition correlated best with the sensory threshold impairment. Hence, older people seem to be more aware of impaired vision and hearing as compared to the other senses. The reason may be that impairment of vision and audition is more obvious to people as they affect different practical aspects of functioning, such as reading or verbal day-to-day interaction. This was reflected in our tests: vision was tested by a reading task and audition in the range of human speech. Low levels of vision and hearing have been associated with loss of functional independence [68,69], communication difficulties [70] and reduced social engagement [71]. Furthermore, a correlation was observed between touch and audition confirming previous literature which indicate cross-modal effects between these two sensory systems [72] as a consequence of similar encoding mechanisms (i.e., mechanical displacements of tissue and frequency-based processing).

Although this study presents new empirical data, some limitations—which call for cautious interpretation—should be outlined. First, sample size may be biased towards people who were more interested in olfaction, as we advertised it from the Smell and Taste Clinic. This limits the generalizability of our findings as responses may differ from the broader population. Second, these data are cross-sectional and therefore do not support any causal conclusions. Longitudinal studies are therefore mandatory to assess the trends of sensory loss for each modality and the interactions among them in relation to different clinical and demographic parameters. A third limitation of the present study was the assessment of only one test for each modality. This was done in order to keep the session relatively short; however, a more fine-grained and comprehensive examination of each modality may reveal different results. Especially interesting in this respect would be the test of daily life functioning for each sense, such as speech perception for audition. Fourth, the failure in demonstrating a common cause could also be rooted in the central processing of sensory stimuli that is much dependent on experiences, training, cognition, and other factors and particularly able to compensate for peripheral changes. Therefore, the sensory decline cannot be entirely explained the peripheral effects as our study design does not allow to disentangle peripheral vs central effects.

Despite the limitations of the research design discussed above, we were able to assess multiple sensory domains–providing a global context for evaluating sensory performance—using a common cross-modality psychophysical testing procedure within the same participants. Understanding the normal age-related decline of sensory thresholds is important because diminished sensory sensitivity is associated with diminished quality of life and loss of independence that need an early assessment.

All in all, this study provides evidence showing differences between sensory threshold and subjective global sensory impairment. Older people seem to be more aware of deficits in vision and audition than they are for any of the other senses and they perceive a sensory association, hence they seemingly expect that impairment in one sense implies impairment in other modalities. However, despite this adverse expectation, our study showed that impairment of one sense does not imply an impairment of the other senses as well. These data convey therefore a positive message to older people and their physicians, assuring them about the fact that loss in one sensory system does not necessarily imply a cascade of loss in the other senses as well.
